# Inbred Mouse Models in *Cryptococcus neoformans* Research

**DOI:** 10.3390/jof10060426

**Published:** 2024-06-17

**Authors:** Minna Ding, Kirsten Nielsen

**Affiliations:** Department of Microbiology and Immunology, University of Minnesota, Minneapolis, MN 55455, USA

**Keywords:** *Cryptococcus neoformans*, inbred mouse models, host immune responses, host–pathogen interactions, cryptococcal meningitis, cryptococcal immune reconstitution inflammatory syndrome, latent *C. neoformans* infection, damage-response framework, fungus, human fungal pathogen

## Abstract

Animal models are frequently used as surrogates to understand human disease. In the fungal pathogen *Cryptococcus* species complex, several variations of a mouse model of disease were developed that recapitulate different aspects of human disease. These mouse models have been implemented using various inbred and outbred mouse backgrounds, many of which have genetic differences that can influence host response and disease outcome. In this review, we will discuss the most commonly used inbred mouse backgrounds in *C. neoformans* infection models.

## 1. Introduction

The use of inbred laboratory mouse strains is essential for broadening our understanding of the host response to Cryptococcus infection. However, many of the common mouse strains that are used for these studies have genetic variations that impact phenotype, especially as it relates to immune response [1]. The accurate interpretation of experimental results requires a comprehensive understanding of the impact a mouse genotypic background has on the overall host response during *C. neoformans* infections. While there are numerous mouse strains that are currently being utilized in the research community, we will focus our discussion on the commonly used inbred strains in *C. neoformans* research: C57BL/6J, A/J, BALB/c, CBA/J, and DBA/2J. We will discuss key phenotypic and genotypic variances in each inbred strain that may affect the host response to *C. neoformans* infection. Finally, we will provide some general recommendations for choosing the appropriate inbred strain(s) for a proposed study.

## 2. Mouse Models of Cryptococcus Disease and Their Relationship to the Damage-Response Framework

**Figure 1:**
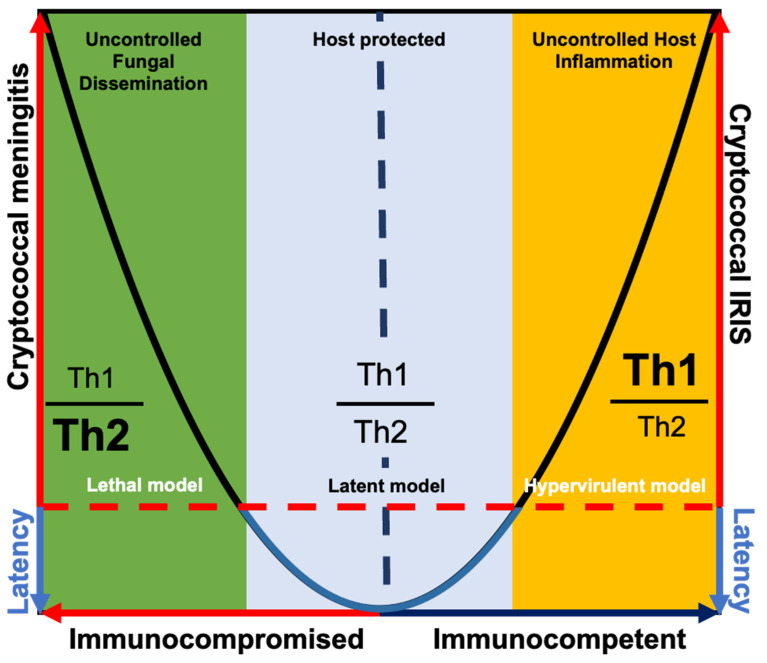
**Damage-response framework parabola in Cryptococcus infections.** Y-axis shows disease outcome and X-axis shows host immune status, whereby a dysregulated immune response and immunocompromised state is hypothesized to promote fungal dissemination (cryptococcal meningitis), and dysregulated Th1 activity and immunocompetent state is hypothesized to promote pathogenic host inflammation or immune reconstitution inflammatory syndrome (cryptococcal IRIS). And, a balanced Th1/Th2 response is hypothesized to control *C. neoformans* infection (latency). Cryptococcal meningitis can be modeled by lethal *C. neoformans* infection [9,10,11,12,13,14,15,16]; latency can be modeled by the latent *C. neoformans* model using clinical isolates [25]; and cryptococcal IRIS can be modeled by either intravenous injection of a high inoculum dose of *C. deneoformans* [17], or adoptive transfer of CD4 T-cells into *C. deneoformans* 1841 [18] or *C. neoformans* H99 [19] infected RAG1-/- mice. Figure adapted from (Pirofski and Casadevall, 2017 [3]; Skipper et al., 2019 [4]).

First proposed by Casadevall and Pirofski in 1999, and then revisited in 2017 for *C. neoformans* specifically, the damage-response framework is a parabola used to predict disease outcome based on an individual’s immune status (Figure 1) [2,3]. In the clinical setting, the damage-response framework is used to manage cryptococcal meningitis in immunocompromised individuals, which is often a delicate balance between antifungal therapy and immune-enhancing therapy [4]. From a research standpoint, we can also use the principles set by the damage-response framework to postulate how the host immune status can drive disease pathogenesis in *C. neoformans*, whereby a dysregulated Th2-dominant response drives fungal dissemination and a dysregulated Th1-dominant response drives rampant inflammation [4,5,6] (Figure 1). However, the pathophysiology behind the progression from latent *C. neoformans* infection to cryptococcal meningitis to cryptococcal immune reconstitution inflammatory syndrome (IRIS) remains limited and reliant on clinical observations [6,7,8].

For many years, researchers relied on a mouse inhalation model of lethal *C. neoformans* infection to study the host and pathogen dynamics in cryptococcal meningitis [9,10,11,12,13,14,15,16]. Additional mouse models were later developed to study other aspects of the cryptococcal damage response framework. To our knowledge, the study of cryptococcal IRIS is limited to only two models; one involving the intravenous injection of a high inoculum dose of *C. deneoformans* 52D into C57BL/6J mice [17] and the second involves the adoptive transfer of CD4 T-cells into *C. deneoformans* 1841 [18] or *C. neoformans* H99 [19] infected RAG1-/- mice. On the other hand, the development of latent mouse models was dependent on genetically engineered *C. neoformans* strains to replicate certain aspects of latent infection, such as an attenuated *C. neoformans* Δ*gcs1* strain to study granuloma formation [20,21] or an H99 strain expressing IFNγ to study fungal clearance [22,23,24]. In recent years, a latent mouse model was also developed using clinical *C. neoformans* isolates [25]. Altogether, these mouse models of Cryptococcus disease allow researchers to interrogate the pathophysiology of *C. neoformans* infection in a comprehensive manner.

## 3. C57BL/6J Inbred Mouse Strain

The C57BL/6J mouse strain originated from the Jackson Laboratory (JAX) and was the first sequenced mouse genome [26,27]. A sub-strain of C57BL/6J mice are C57BL/6N mice, which originated from C57BL/6J breeders that were shipped to the National Institutes of Health [26]. There are key genetic and phenotypic differences between C57BL/6J and C57BL/6N mice that make it inadvisable to use both interchangeably [28,29,30]. One notable aspect of C57BL/6J mice is their impaired glucose tolerance, making this strain a good model for studying type 2 diabetes [31]. These mice also have a high susceptibility to diet-induced obesity and atherosclerosis [32]. In comparison to the other inbred mouse strains discussed here, C57BL/6J mice have an intact complement pathway [33], and have a full repertoire of neuronal apoptosis inhibitory proteins (NAIPs) that are involved in anti-apoptosis, phagocytosis, and inflammasome activation [34] (Table 1). Finally, C57BL/6J mice are also prone to hereditary hydrocephalus [35], which can be a potential confounding factor for *C. neoformans* infection models. We recommend checking brain fungal burden or blood cryptococcal antigen titers [25] in all C57BL/6J mice infected with *C. neoformans* that develop a domed head (or focal neurological symptoms) to confirm cryptococcal meningitis.

Historically, the underlying immune background of the C57BL/6J strain was thought to be Th1-skewed based on its splenocyte response to Concanavalin A (Con A) [36]. However, those initial findings may have contributed to an over-generalization of the host response against diverse antigenic insults. For instance, in the allergic airway disease model, C57BL/6J mice challenged and sensitized with ovalbumin (OVA) can generate a Th2-skewed response, albeit weaker compared to that of BALB/c mice [37]. Indeed, the host immune response in C57BL/6J mice is highly dependent on *C. neoformans* strain-specific differences. One study found that C57BL/6J mice intratracheally infected with *C. deneoformans* 52D had a Th2-skewed immune response with an increased production of cytokines associated with the Th2 phenotype, pulmonary eosinophilia, and elevated serum IgE [38]. Similarly, *C. neoformans* laboratory strains, such as KN99α, also elicit a Th2-dominant response in C57BL/6J mice in the lethal model [10,11]. However, in the latent *C. neoformans* infection model, C57BL/6J mice infected with the clinical isolate UgCl223 were able to survive for prolonged periods of time, contain yeast cells within pulmonary granulomas, and had a Th1-skewed immune response [25]. Thus, C57BL/6J mice are a unique mouse strain that has a Th2-skewed response to lethal *C. neoformans* infections, but a Th1-skewed response to latent *C. neoformans* infections (Table 2).

Of note, vaccine discovery and development studies have observed that the C57BL/6J mouse strain has a tendency of generating a milder inflammatory response against *C. neoformans* compared to other mouse strains. C57BL/6J mice vaccinated with a *C. neoformans cda1*Δ*2*Δ*3*Δ chitosan-deficient strain have demonstrably reduced protection against *C. neoformans* KN99α infection compared to BALB/c, A/J, and CBA/J mice [39], even though unvaccinated C57BL/6J mice and BALB/c mice have comparable median survival times when infected with *C. neoformans* KN99α [40]. The same phenomenon is also observed with glucan particle-based vaccines, where the efficacy against *C. neoformans* KN99α infection in C57BL/6J mice is lower compared to BALB/c mice [41]. While these findings suggest that the inflammatory response against *C. neoformans* infection in the C57BL/6J mouse strain may be less robust compared to the BALB/c mouse strain, the implications of this phenomenon are unclear. Regardless, based on their well-characterized genetic background and diverse repertoire of available immunological tools, the inbred C57BL/6J mouse strain remains an ideal model for studying the host immune response against *C. neoformans* infection (Table 2).

## 4. A/J Inbred Mouse Strain

A/J mice are a widely used model in cancer and immunology research and were the first inbred mouse strain used for the *C. neoformans* inhalation infection model [16]. Unlike C57BL/6J mice, A/J mice are resistant to diabetes and obesity, exhibiting both insulin resistance and glucose intolerance [42,43]. Since A/J mice are dysferlin-deficient, they are also a suitable model for studying dysferlinopathies, such as Duchenne muscular dystrophy [44,45]. From a pulmonary standpoint, A/J mice are uniquely susceptible to lung tumorigens, such as those found in tobacco smoke, making them an ideal model for studying lung tumorigenesis [46,47]. In addition, A/J mice are highly reactive to methacholine in the absence of antigen sensitization and airway challenge, and have been used to study the naïve airway hyperresponsiveness that is associated with asthma development [48]. Interestingly, A/J mice that are sensitized and challenged with sheep red blood cells or OVA prior to methacholine challenge will generate a Th2-skewed pulmonary immune response [49]. The most unique aspect of A/J mice are their genetic background, in that they have a loss-of-function frameshift mutation in their complement component 5 (C5) (*Hc^0^*) [33]. In addition, these mice have a defective NAIP5 allele (*Naip5*) and are resistant to anthrax lethal toxin (*Nlrp1b^R/R^*) [34] (Table 1). Altogether, these aspects make the inbred A/J mouse strain an enticing model for studying early innate immune responses.

Although the immune polarization of A/J mice in response to *C. neoformans* infection is not well characterized (Table 2), many of the immunology studies that use A/J mice have capitalized on their C5 complement deficiency. The majority of published studies using A/J mice have focused on the protective effect of monoclonal antibodies against *C. neoformans* infection [50,51,52,53,54,55], vaccine discovery with a heat killed *C. neoformans fbp1*Δ F-box protein-deficient strain [56,57], a heat killed *C. neoformans cda1*Δ*2*Δ*3*Δ chitosan-deficient strain [39], and glucuronoxylomannan-protein conjugates [58]. From a host response perspective, an early study looking at survival characteristics of different inbred mouse strains categorized A/J mice as “sensitive” following intravenous infection with *C. deneoformans* B3502 [59]. In comparison to other inbred mouse strains including C57BL/6J and BALB/c mice, A/J mice also seem to have poor antibody responses to glucuronoxylomannan (GXM) compared to other inbred mouse strains, possibly suggesting that the humoral response generated against *C. neoformans* infection is different in A/J mice [58]. That being said, the efficacy of different monoclonal antibody isotypes against GXM is comparable in terms of survival for both A/J and C57BL/6J mice during *C. neoformans* infection [53]. Overall, these findings demonstrate the utility of the inbred A/J mouse strain in *C. neoformans* vaccination development.

Recently, a study used A/J mice to assess differential virulence in *C. neoformans* clinical isolates and found that the inhalation infection model in A/J mice was able to recapitulate patient outcomes [60]. A/J mice infected with different clinical isolates also had differential median survival rates, showing that disease outcome in A/J mice depends on *C. neoformans* strain-specific genotype. Thus, A/J may be ideal models for elucidating *C. neoformans* virulence factors, as evidenced by studies on melanin production [61] and antiphagocytic proteins [62]. We recommend that A/J mice be used to study the influence of the Cryptococcus genotype on disease outcomes, especially given their ability to recapitulate patient outcomes (Table 2).

## 5. BALB/c Inbred Mouse Strain

BALB/c mice are well-known for their ability to form plasmacytomas following mineral oil injection, which is used in the production of monoclonal antibodies [63]. In addition, BALB/c mice are predisposed to dystrophic cardiac calcinosis, which is the mineralization of cardiac tissue, and are used as a model to study rare calcinotic diseases in humans [64]. Studies also found that BALB/c mice are susceptible to murine encephalomyelitis virus-induced demyelinating disease [65]. From a pulmonary standpoint, BALB/c mice are useful asthma models in antigen challenge experiments and develop a Th2-skewed response [36,37,66].

Like with C57BL/6J mice, the immune response to *C. neoformans* infection has been extensively studied in BALB/c mice. When infected with *C. neoformans* H99, BALB/c mice develop a Th2-skewed pulmonary immune response with elevated Th2-associated cytokine production [13,67]. However, when infected with *C. deneoformans* 52D, BALB/c mice are able to clear the infection and develop a Th1-skewed immune response with increased production of cytokines associated with the Th1-phenotype, decreased IL-10 production, and no significant elevation in systemic IgE production [38,68]. In addition, the fungal burden in BALB/c mice infected with *C. deneoformans* 52D is significantly lower compared to C57BL/6J mice, and these findings seem to correlate with an increased lymphocytic CD4 response in BALB/c mice versus an increased granulocytic eosinophil response in C57BL/6J mice [38]. It is intriguing that *C. deneoformans* infection in C57BL/6J mice causes a Th2-skewed response, whereas the same infection in BALB/c mice results in a Th1-skewed response. Thus, we can infer from the BALB/c mouse inhalation and intratracheal infection models that Cryptococcus virulence and host survival outcome are dependent on both host and pathogen-related factors.

One aspect of host immunity against *C. neoformans* in BALB/c mice that is well-studied is IL-33 and its receptor ST2. IL-33 is a pleiotropic cytokine that is primarily produced by alveolar epithelial cells during *C. neoformans* and *C. deneoformans* infection [69]. In BALB/c mice, the IL-33 receptor ST2 is responsible for driving the Th2 response and is required for IL-5 and IL-13 production [12,70,71]. In addition, regulatory CD4 T-cells (Tregs) expressing ST2 are immunosuppressive and accumulate during the early phases of *C. deneoformans* 52D infection (up to 7 days post-infection), but are then replaced by more inflammatory Tregs as the infection progresses (past 14 days post-infection) [72]. Interestingly, these findings were only partially replicated in C57BL/6J mice, where only IL-33 deficiency, but not ST2 deficiency, results in decreased fungal burden and a reduction in Th2 cytokines during *C. neoformans* H99 infection [73]. While the differences in host response between BALB/c and C57BL/6J mice are likely multifactorial, these studies suggest that IL-33 and its receptor ST2 may be a key factor in driving the differential host response between BALB/c and C57BL/6J mice and should be considered when choosing an appropriate inbred mouse model.

From a genetic standpoint, BALB/c mice are C5 complement sufficient, are sensitive to anthrax lethal toxin (*Nlrp1b^S/S^*), and do not have any known deficiencies or mutations associated with the NAIP complex [33,34] (Table 1). Overall, the immune response to different *Cryptococcus* species in BALB/c mice seems to depend on the host genetic background and pathogen strain genotype. Like with C57BL/6J mice, BALB/c mice are an ideal strain for studying the host immune response against different *C. neoformans* strains, and may serve as a useful juxtaposition against C57BL/6J mice when delineating host-specific versus pathogen-specific factors that affect Cryptococcus virulence (Table 2).

## 6. CBA/J Inbred Mouse Strain

When immunized with thyroglobulin emulsified in complete Freund’s adjuvant, CBA/J mice develop experimental thyroiditis and are a well-characterized model for Hashimoto’s disease or granulomatous experimental autoimmune thyroiditis (G-EAT) [74,75]. It has been previously shown that G-EAT progression is characterized by a pro-inflammatory cytokine response and G-EAT resolution required IL-10 [76]. CBA/J mice are also good models for studying hearing and deafness, as they develop hearing loss as they age [77].

In CBA/J mice, Cryptococcus infection results in somewhat conflicting findings. In one study, CBA/J mice were less resistant to intratracheal *C. deneoformans* 52D infection (at starting inoculums of 10^5^ or 10^6^ colony forming units (CFUs)) compared to BALB/c mice and infected CBA/J mice had higher brain fungal burden, and lower serum IgM and IgG in response to GXM [68]. However, in a separate study, CBA/J mice were more resistant to intratracheal *C. deneoformans* 52D infection when infected with a lower 10^4^ CFU inoculum dose compared to BALB/c mice, with diminished eosinophil recruitment [78]. These findings were also corroborated in another study where the intratracheal infection of CBA/J mice with *C. deneoformans* 52D (at the same 10^4^ CFU inoculum dose) resulted in prolonged survival, pulmonary neutrophilia, and increased IFNγ and IL-17 production in the lungs [79]. These somewhat conflicting results suggest that virulence outcomes in CBA/J mice may be dose-dependent and is based on the starting inoculum dose of *C. deneoformans*. Interestingly, CBA/J mice intranasally infected with *C. neoformans* KN99α at a starting inoculum dose of 10^5^ CFUs also have a shorter median survival time compared to C57BL/6J and BALB/c mice infected with 10^4^ CFUs of KN99α [40]. Although, the decreased median survival in CBA/J mice could also simply be due to the higher inoculum dose of *C. neoformans* KN99α compared to C57BL/6J and BALB/c mice. Overall, these data seem to suggest that CBA/J mice have a Th1-polarized response against *C. deneoformans* 52D infection when infected intratracheally at 10^4^ CFUs (Table 2).

In terms of genetic polymorphisms, CBA/J mice are C5 complement sufficient, sensitive to anthrax lethal toxin (*Nlrp1b^S/S^*), and do not have any known deficiencies or mutations associated with the NAIP complex [34] (Table 1). Interestingly, researchers have identified three chromosomal regions in CBA/J mice, *Cnes1-3*, which are associated with lung *C. deneoformans* 52D fungal control. In addition, the insertion of the CBA/J *Cnes2* chromosomal region into C57BL/6J mice results in a Th1-skewed response [80,81]. In a similar study, another chromosomal region in CBA/J mice, *Cnes4*, confers resistance to *C. deneoformans* 52D infection [79]. However, it is still unclear what gene(s) within the *Cnes2* or *Cnes4* regions are responsible for resistance against Cryptococcus infection and the mechanisms by which these regions promote a Th1-skewed response. Given its intrinsic resistance against *C. deneoformans* 52D infection (albeit at a specific inoculum dose of 10^4^ CFUs), the CBA/J mouse strain could be used to study host resistance versus susceptibility against other *C. neoformans* strains (Table 2). In addition, CBA/J mice might be an ideal model for studying the damage-response framework, as evidenced by the differential host response that is dependent on the starting inoculum dose for the same *C. deneoformans* strain.

## 7. DBA/2J Mouse Strain

DBA/2J mice are one of the oldest inbred strains of mice, and are widely used in cardiovascular biology, neurobiology, and sensorineural research. The DBA/2J strain is also an ideal model for glaucoma research, due to two mutations in *Gpnmb* and *Tyrp* genes for iris pigment dispersion and iris stromal atrophy, respectively, which results in the age-related elevation of intraocular pressure and development of glaucomatous pathology in about 70% of mice [82]. Like in the other inbred mouse strains discussed in this review, the underlying immune polarization of DBA/2J mice was also derived from an early study that demonstrated a mixed Th1/Th2 phenotype based on splenocyte response to Con A [36]. In terms of the pulmonary immune milieu, DBA/2J mice seem to have an increased susceptibility to influenza virus compared to C57BL/6J mice, with increased pro-inflammatory cytokine production and increased viral replication in respiratory cells [83,84,85]. The implications of these findings on Cryptococcus infection in DBA/2J is unknown.

Out of all the inbred mouse strains discussed here, DBA/2J mice are the least characterized in terms of host response to Cryptococcus infection. An early study looking at survival characteristics of different inbred mouse strains categorized DBA/2J mice as “sensitive” following intravenous infection with *C. deneoformans* B3502 [59]. Like A/J mice, DBA/2J mice are C5 complement deficient (*Hc^0^*) and are resistant to anthrax lethal toxin (*Nlrp1b^R/R^*) [33]. However, DBA/2J mice are similar to C57BL/6J mice in that they have no known NAIP deficiencies or mutations [34] (Table 1). As with A/J mice, the majority of studies utilize DBA/2J mice to elucidate the role of C5 complement in the host immune response [86]. From that perspective it is unsurprising that DBA/2J, like A/J mice, are “poor responders” with low antibody titers against GXM [87]. Although, unlike A/J mice, DBA/2J mice treated with monoclonal antibodies against GXM are more resistant to *C. neoformans* infection compared to BALB/c and C57BL/6J mice [88]. Interestingly, DBA/2J mice treated with a monoclonal antibody specific to GXM do not recruit phagocytes to the lung, whereas C5-sufficient BALB/c mice did have local phagocytic recruitment during *C. neoformans* infection [89]. Furthermore, a separate study found that peritoneal macrophages isolated from DBA/2J mice had poor phagocytic and fungistasis activity against Cryptococcus yeast cells [90]. These findings suggest that the early innate response to *C. neoformans* infection in DBA/2J mice is dependent on the C5 complement pathway, but can be overcome by antibody-mediated opsonization [91].

Overall, the DBA/2J mouse strain could serve as a useful comparison to A/J in future *C. neoformans* clinical isolate studies, since the replication of disease and immune phenotypes in DBA/2J mice compared to A/J mice would confirm that complement and/or *Nlrp1b^R/R^* resistance to anthrax lethal toxin is associated with *C. neoformans* virulence (Table 2).

## 8. Recommendations for Inbred Mouse Strains Based on *C. neoformans* Immunology Studies Published since 2015

Since 2015, the majority of murine model studies on innate and adaptive immunity in *C. neoformans* (and *C. deneoformans*) infection have utilized C57BL/6J mice (Table 3). In contrast, the number of studies using BALB/c mice are comparatively smaller and focus mainly on innate immunity (Table 3). In addition, BALB/c and CBA/J mice have been popular models in recent years for vaccine development and discovery, perhaps due to the relatively stronger protective response against *C. neoformans* following vaccination compared to C57BL/6J mice [39] (Table 3). DBA/J mice seem to have largely fallen out of favor for studying the host inflammatory response (Table 3). Based on these trends, the C57BL/6J mouse strain remains a popular choice for the study of the host response against *C. neoformans* infection. However, the A/J, BALB/c, CBA/J, and even DBA/J mouse strains can serve as a useful tool for identifying host factors that are absent or minimally elicited in C57BL/6J mice.

When choosing an appropriate mouse model, we recommend that researchers closely align the key characteristics of each inbred mouse strain with the goals of their proposed study. Compared to all other inbred mouse strains, the C57BL/6J mouse strain is the most well-characterized in terms of host response against *C. neoformans* infection. Therefore, proposed studies on the host immune response, especially those that aim to overturn established dogma in the Cryptococcus field, should use the C57BL/6J strain, or include it in addition to other models. The host response of the BALB/c strain against *C. neoformans* is the second-most well characterized and this strain can often elicit a stronger inflammatory response against *C. neoformans* compared to C57BL/6J mice. We would also recommend the BALB/c strain for studying the host immune response against *C. neoformans* infection, and this strain should be considered especially if researchers have difficulty eliciting a strong inflammatory response in C57BL/6J mice. Historically, vaccine discovery and development studies have used multiple inbred mouse strains to demonstrate efficacy. Based on the differential immune polarization of the inbred mouse strains discussed in this review, we would continue to recommend at least two inbred mouse strains be used in the initial characterization of vaccines or immunization strategies against *C. neoformans*. Proposed studies that aim to compare host response between different clinical or environmental *C. neoformans* isolates could consider using A/J mice, as this inbred mouse strain has been found to reliably recapitulate patient outcomes. Although the use of the DBA/2J mouse strain in *C. neoformans* research has declined significantly, its unique genetic background may yield useful information when used in conjunction with other inbred mouse strains. Finally, we recommend that all future Cryptococcus studies provide appropriate rationale for (1) mouse strains, (2) *C. neoformans* strains, (3) starting inoculum dose, and (4) mode of infection (i.e., intranasal vs. intratracheal vs. intravenous vs. intraperitoneal).

## 9. Outbred Mouse Strains

Outbred mouse strains are known for their genetic heterozygosity and variability and are thought to be a better mimic of the variability seen in human populations. Many outbred mouse strains have been derived; two of the more commonly used strains in Cryptococcus research are the OF1 and CD-1 strains. The CD-1 strain is derived from Lynch’s Swiss mice [165]. The OF1 strain is derived from the CF-1 outbred stock (which is not descended from Swiss mice) and subsequently acquired by Charles River Laboratories France, hence the name Oncine France 1 (OF1) [166]. Both the CD-1 and OF1 mouse strains have been historically used to study a wide range of host- and pathogen-specific aspects of *C. neoformans* virulence, including immunology, pathophysiology, pharmacology, and toxicology.

While these studies have not yet been attempted in Cryptococcus research, outbred mouse strains could potentially be used to selectively breed for phenotypes of interest, such as a specific type of immune response or increased resistance against infection [167]. Unlike inbred mouse strains, outbred mouse strains also have polymorphic loci and are ideal models for QTL mapping which could allow researchers to identify gene alleles that affect survival versus susceptibility against *C. neoformans* infection [167,168]. Thus, the value that outbred mouse strains bring to Cryptococcus research lies in their ability to parse out the complex host genetic networks that influence pathogenesis, rather than the characterization of host immune phenotypes that are easily affected by minute changes in the host genetic background.

Therefore, we recommend extreme caution in using outbred mice to study the immune response against *Cryptococcus* species or strains. Outbred strains by definition have labile genetic backgrounds and the genotype of each individual mouse in a study is frequently unknown [167], thus it would be extremely difficult to reproduce and precisely characterize the host immune response to specific *C. neoformans* strains. Outbred strains are also at an increased risk for genetic drift and bottleneck events, requiring stringent breeding schemes to maintain an appropriate degree of genetic heterogeneity [169]. It is also important to note that there are no standard genetic quality control methods for maintaining outbred mouse colonies; commercial breeders often do not publish their methods for monitoring genetic diversity in their outbred stocks and institutional outbred colonies are subject to a high degree of variability in quality control [167].

Most critically, outbred strains require larger sample sizes in order to generate enough statistical power compared to equivalent inbred mouse studies [167]. As such, the physiological relevance of outbred strains to human genetic diversity should not be used as the sole argument for their inclusion into an experimental study. Instead, many researchers propose that an experimental study utilizing more than one inbred strain and a factorial experimental design would require a smaller sample size and yield equivalent statistical significance compared to a study with a single outbred strain [167,170]. Thus, it would be cost-prohibitive, time-consuming, and unethical to use outbred strains without appropriate rationale, stringent experimental design, and the careful monitoring of mouse colonies.

## 10. Conclusions

While the C57BL/6J, A/J, BALB/c, CBA/J, and DBA/2J inbred mouse strains are used extensively in Cryptococcus research, many of these mouse strains have yet to be fully characterized in the context of *C. neoformans* infection. Thus, it is difficult to draw definitive conclusions between studies that are performed with different mouse backgrounds, especially as it relates to the host immune response. However, compared to outbred mouse strains, inbred mouse strains are a more ideal model for studying the host response to *C. neoformans* infections because the genetic homozygosity of these strains allows researchers to better control for complex host variables.

## Figures and Tables

**Table 1:** Genetic mutations associated with immune response in C57BL/6J, A/J, BALB/c, CBA/J, and DBA/2J mice.

Strains	Complement C5	NOD-like Receptor Proteins	Neuronal Apoptosis Inhibitory Proteins (NAIPs)
**C57BL/6J**	Sufficient	*Nlrp1b^R/R^*—resistant to anthrax lethal toxin	Intact *Naip1*, *Naip2*, *Naip5*, and *Naip6*
**A/J**	*Hc^0^*	*Nlrp1b^R/R^*—resistant to anthrax lethal toxin	Defective *Naip5* allele
**BALB/c**	Sufficient	*Nlrp1b^s/s^*—anthrax lethal toxin induces caspase-1 and macrophage lysis	No known deficiencies/mutations; intact *Naip4*
**CBA/J**	Sufficient	*Nlrp1b^s/s^*—anthrax lethal toxin induces caspase-1 and macrophage lysis	No known deficiencies/mutations
**DBA/2J**	*Hc^0^*	*Nlrp1b^R/R^*—resistant to anthrax lethal toxin	No known deficiencies/mutations

**Table 2:** Immune polarization against *C. neoformans* and *C. deneoformans* in C57BL/6J, A/J, BALB/c, CBA/J, and DBA/2J mice and recommendations for future studies.

Strains	Immune Polarization against *C. neoformans*	Immune Polarization against *C. deneoformans*	Relative Survival When Infected with *Cryptococcus* Strains *	Recommended Studies
**C57BL/6J**	Th2 polarized response against **KN99α**	Th2 polarized response against **52D**	Survival comparable to BALB/c against **KN99α** at 10^4^ CFUs **	Best for studying the host immune response against different *Cryptococcus* strains
	Th1 polarized response against **UgCl223**		More susceptible to **52D** at 10^4^ CFUs compared to BALB/c	
**A/J**	Unknown	Unknown	“Sensitive” to survival against **B3502** at 5 × 10^6^ CFUs intravenously	Best for studying the influence of Cryptococcus virulence and genotype on disease outcome
**BALB/c**	Th2 polarized immune response against **H99**	Th1 polarized against **52D**	Comparable to C57BL/6J and BALB/c against **KN99α** at 10^4^ CFUs	Best for studying the host immune response against different *Cryptococcus* strains
			More resistant compared to C57BL/6J against **52D** at 10^4^ CFUs	
**CBA/J**	Unknown	Th1 polarized against **52D** at 10^4^ CFUs	More susceptible compared to C57BL/6J and BALB/c against **KN99α** ***	Best for studying host genes that confer resistance versus susceptibility against *Cryptococcus* strains
			More resistant compared to BALB/c against **52D** at 10^4^ CFUs	
			More susceptible compared to BALB/c against **52D** at 10^5^ CFUs	
**DBA/2J**	Unknown	Unknown	“Sensitive” to survival against **B3502** at 5 × 10^6^ CFUs intravenously	Best for comparison against A/J mice to isolate host-specific factors contributing to Cryptococcus virulence

* Unless otherwise stated, all infections were performed either intranasally or intratracheally. ** When appropriate, colony forming units (CFUs) were provided for comparison. *** CBA/J mice were infected with 10^5^ CFUs, but C57BL/6J and BALB/c mice were infected with 10^4^ CFUs.

**Table 3:** Murine infection studies on host immunity in *C. neoformans* and *C. deneoformans* published since 2015.

Strains	Innate Immune Response *	Adaptive Immune Response *	Other
**C57BL/6J**	**43 Publications** [73,92,93,94,95,96,97,98,99,100,101,102,103,104,105,106,107,108,109,110,111,112,113,114,115,116,117,118,119,120,121,122,123,124,125,126,127,128,129,130,131,132,133]	**8 Publications** [9,10,11,17,134,135,136,137]	**18 Publications** [18,19,20,25,39,41,56,57,81,138,139,140,141,142,143,144,145,146]
**A/J**	**0 Publications**	**0 Publications**	**3 Publications** [39,56,57]
**BALB/c**	**9 Publications** [69,71,72,147,148,149,150,151,152]	**1 Publication** [153]	**15 Publications** [18,39,40,41,56,138,142,143,154,155,156,157,158,159,160]
**CBA/J**	**1 Publication** [161]	**0 Publications**	**11 Publications** [20,39,40,56,81,140,144,145,162,163,164]
**DBA/2J**	**0 Publications**	**0 Publications**	**0 Publications**

* Studies were identified via PubMed using the key terms “(Cryptococcus neoformans) AND (mouse OR murine) AND (immune or immunology)”. Of the 1300 results, only peer-reviewed primary research papers published in 2015 or later that used C57BL/6J, A/J, BALB/c, CBA/J, or DBA/2J inbred mouse strains for host immunity studies were included. Studies that used hybrid mice (cross of two inbred strains) or had ambiguous characterization of inbred sub-strains were excluded.
